# Native tissue repair of the female pelvic floor: A four‐level surgical concept

**DOI:** 10.1002/ijgo.16109

**Published:** 2024-12-22

**Authors:** Michal Otcenasek, Katarzyna Borycka, Hynek Herman

**Affiliations:** ^1^ Department of Urology Third Medical Faculty Prague Czech Republic; ^2^ Third Medical Faculty Charles University Prague Czech Republic; ^3^ Department of Colorectal, General and Oncological Surgery Center of Postgraduate Medical Education Warsaw Poland; ^4^ Institute for the Care of Mother and Child Prague Czech Republic

**Keywords:** anatomy, birth trauma, endopelvic fascia, incontinence, obstetric anal sphincter injury, pelvic organ prolapse, proctology

## Abstract

This review describes our experience with native tissue repair of the visceral pelvic fascia, the perineum, and anal sphincters in women. We propose that complex repair of the pelvic floor should consider vaginal support in all three anatomical Delancey's levels, together with more caudal structures—the external and internal anal sphincters. Original illustrations were created to facilitate the understanding of the complex anatomy of common multi‐level defects. As the integrity of connective tissue adds to various aspects of the delicate function of the female pelvic floor, it is complete and as perfect as possible repair is a common goal of both gynecologists and colorectal specialists.

## Synopsis

Practical illustrated surgical anatomy of female pelvic floor, from vaginal apex to anal verge.

## 1 INTRODUCTION

**FIGURE 1:**
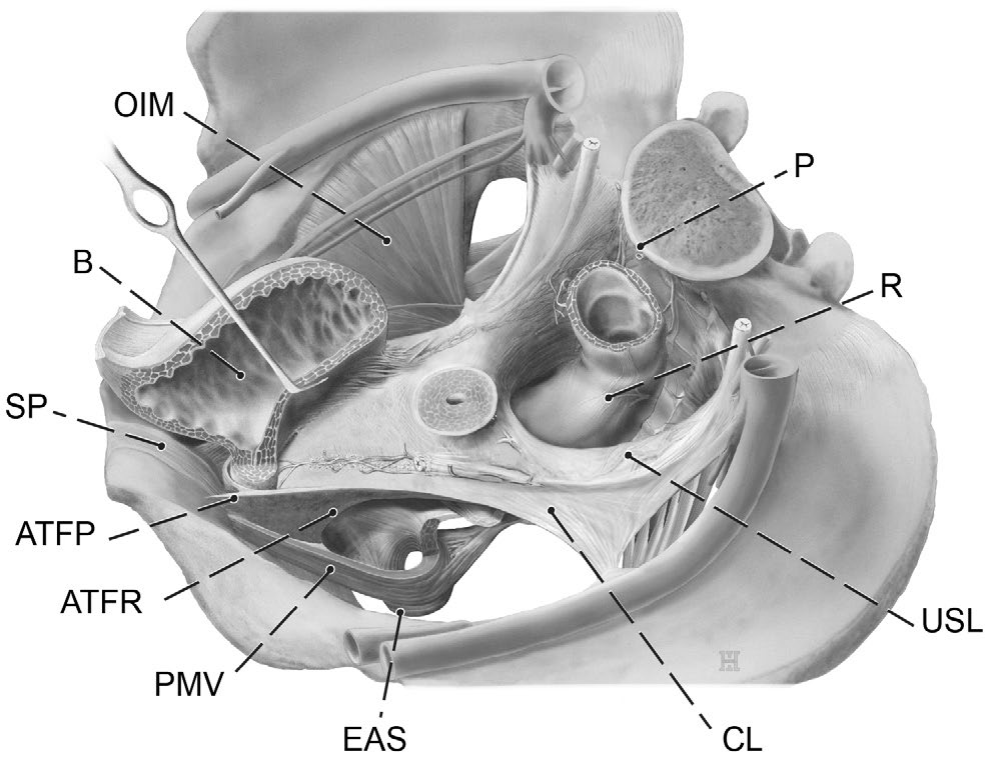
Visceral pelvic fascia and related structures. Three‐dimensional (3D) left upper view. ATFP, tendinous arch of pelvic fascia; ATFR, tendinous arch of rectovaginal fascia; B, urinary bladder; CL, cardinal ligament; EAS, external anal sphincter; OIM, obturator internus muscle; P, promontory; PVM, pubovisceral muscle; R, rectum; SP, symphysis pubis; USL, uterosacral ligament.

**FIGURE 2:**
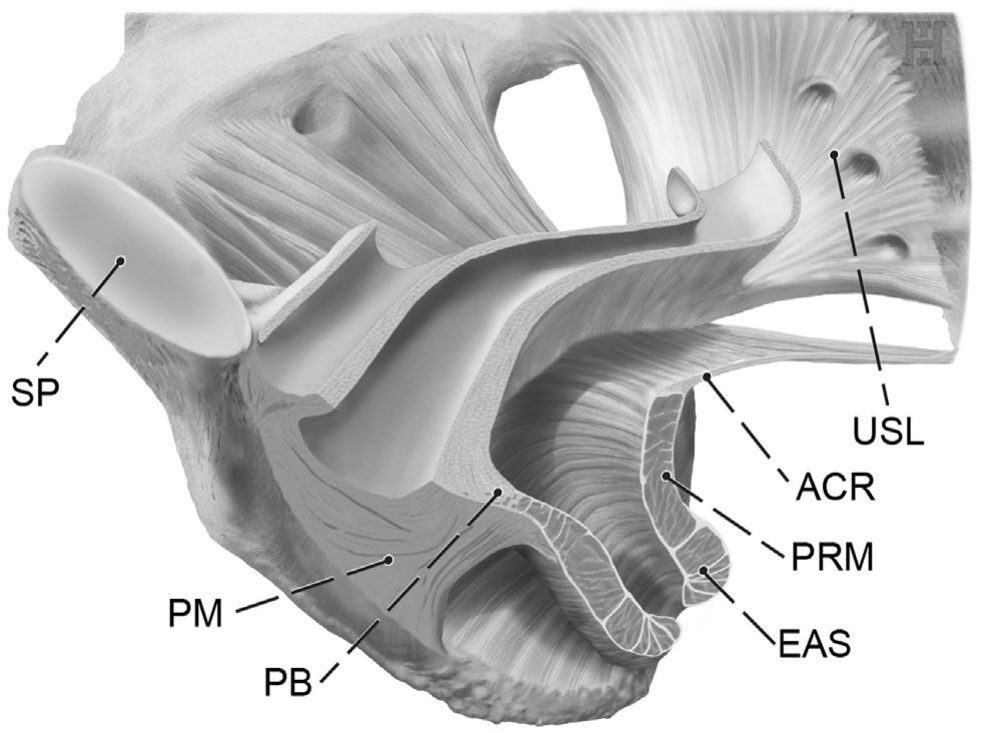
Connective tissue of the female pelvic floor. Inferior view. The major target structures of vaginal reconstructive surgery are shown. The visceral pelvic fascia, perineal membrane, and external anal sphincter are indicated in relation to the levator ani muscle and parietal pelvic structures. ACR, anococcygeal (or iliococcygeal) raphe; EAS, external anal sphincter; PB, perineal body; PM, perineal membrane; PRM, puborectal muscle; SP, symphysis pubis.

Pelvic floor defects are a significant source of female morbidity worldwide.^1^ Their complex nature makes treatment challenging, and their outcomes vary widely.^2^ The expectations that have been placed on transvaginal synthetic materials have not been met, due to the unacceptably high risk of serious complications.^3^ Thus, vaginal/transperineal repair using native tissue for reconstruction is one of two primary options for surgeons, together with abdominal repair (open, laparoscopic or robotic). The principal surgical approach that uses the patient's native structures is to expose the relevant connective tissue, repair the defects, and reinforce their laxity with sutures. A thorough understanding of the normal and pathologic anatomy of the pelvic floor and the function of its structures is paramount. The aim of the present study was to identify the surgically pertinent structures and define how their defects should be repaired. The visceral pelvic fascia, perineal membrane, and external anal sphincter muscle are considered a single, combined entity and should be managed as such (Figures 1, 2, and 6).^4^ This is particularly important when performing repairs after childbirth. The sphincter ani muscle is rarely torn alone, almost always the trauma includes both perineal body and the sphincter, very often together with the rectovaginal fascia.

## 2 SUPPORT LEVELS

The well‐established anatomical consensus describes three levels of the visceral pelvic fascia and perineum about their support of the vagina.^5^ Level I suspends to the vaginal apex (uterosacral and cardinal ligaments). Level II consists of the connection of the visceral pelvic fascia to the superior fascia of the levator ani muscle (parietal pelvic fascia). Level III is defined as the direct attachment of the vagina to the perineal membrane and adjacent superficial perineal space. For practical reasons, the inclusion of the anal canal structures to create a robust surgical scheme appears to be justified due to their anatomical proximity to the perineal body and the fact that the sphincters are not isolated and because its tears elongate to the perineal body and further to the rectovaginal fascia in most cases (Figure 5). Thus, any repair in this area should be planned and performed with attention being paid to all involved structures. The designation of the sphincter ani muscle and anal canal as surgical level IV of the female pelvic floor is simple and pertinent.

### 2.1 Defects

Defects of the pelvic floor include tearing and stretching. Tears of the muscle or connective tissue occur primarily due to vaginal childbirth. Stretching of connective tissue generally results from chronic overload and develops gradually over time. Stretching can also result from vaginal childbirth or can develop gradually due to chronic overload later in life. In most cases, our patients have a combination of one or several tears and secondary elongation of overloaded tissue.

### 2.2 Surgery

The initial step in the surgical plan is to map the tissue defects. The position, shape, and symmetricity of the apical vault, the presence or absence of lateral vaginal sulci, and indentations at points of insertions provide clues about the integrity of paravaginal connective tissue. No preserved lateral structure should be missed because incorporation into our reconstruction enhances its future stability. Temporary stitches can be placed at the start of surgery to facilitate its identification after opening of the spaces. The absence of normal vaginal rugae indicates central stretching of the fascia. The shape and symmetricity of the introitus and the presence of scars improve our understanding of the true extent of the defect.^4^ Palpation of the muscles vaginally, the extent of the rectocele, and the function of the sphincter ani rectally, in combination with two‐dimensional (2D)/three‐dimensional (3D) ultrasonography, further help us understand the scope and nature of the problem.^6^

**FIGURE 3:**
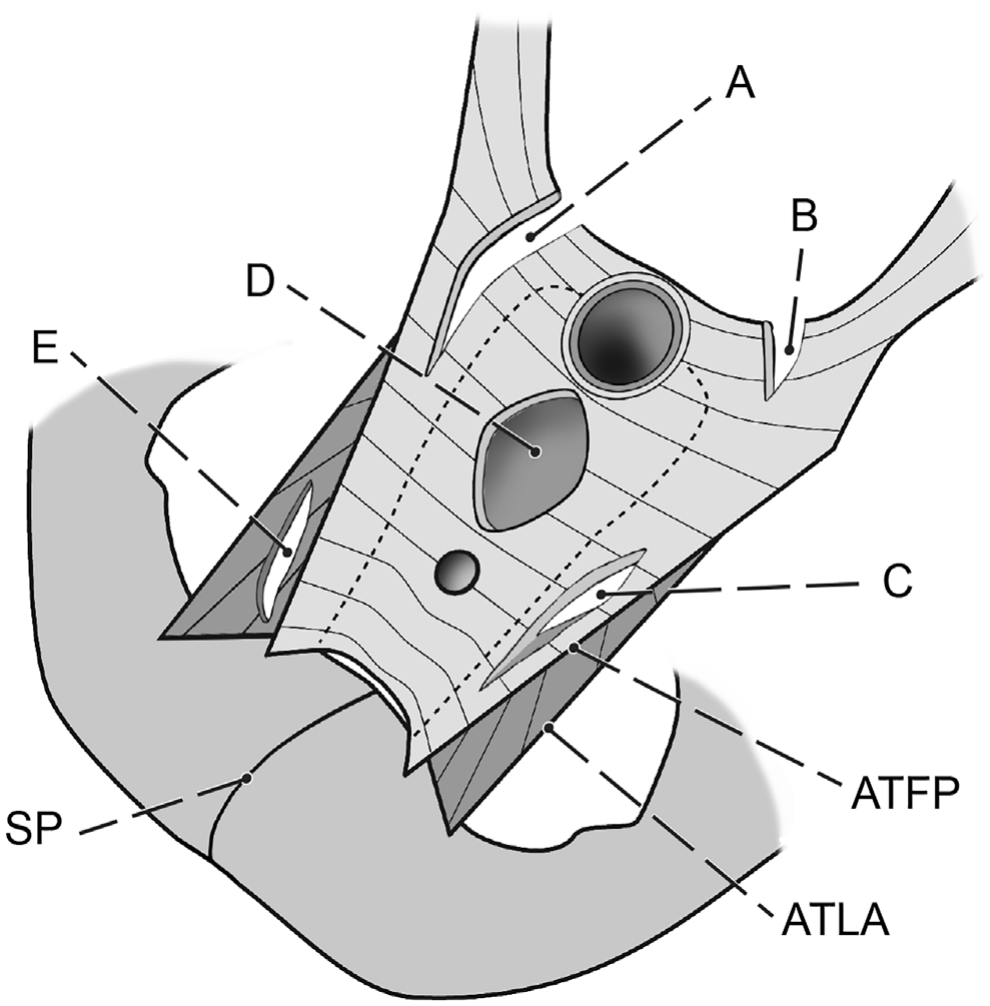
Location of common defects of the visceral pelvic fascia and levator ani muscle. (A) Lateral defect of level I and II (uterosacral ligament, cardinal ligament and vesicovaginal fascia. (B) Shallow defect of the level I. (C) Lateral defect of the level II (paravaginal defect). (D) Central (pulse type) defect of the level II. (E) Lateral defect of the muscle (avulsion). ATFP, tendinous arch of pelvic fascia; ATLA, tendinous arch of levator ani; SP, symphysis. Extent and combination of defects vary individually.

However, a precise, complex, objective description is rarely possible, and its interpretation will depend on the surgeon's experience. Figures 3 and 5 show typical defect locations.

The first step during vaginal reconstruction is to expose the connective tissue by opening the vaginal or perineal skin. Old scars are mostly removed. The defects do not always lie on the midline (in point of fact, the opposite is true). The exposure should be sufficiently large to reach the lateral parts with an intact connection to parietal structures. Then, if a defect in connective tissue can be identified, its edges are approximated. The elongated tissue is then shortened and reinforced by plication through vertical or horizontal mattress sutures. By addressing all levels, primarily I and III, the vaginal axis is restored, allowing for normal pressure distribution and avoiding excessive load on connective tissue.

### 2.3 Level I

The cornerstone of a successful repair is to identify the remaining suspensory part of the visceral pelvic fascia (uterosacral and cardinal ligaments). Joining the left and right sections of this apparatus lifts the apex (a double‐latch mechanism) and directs it retrograde. Thus, the vaginal apex is relocated to its original position above the levator ani muscle (away from the introitus), where it is protected from the excursions of intra‐abdominal pressure.

**FIGURE 4:**
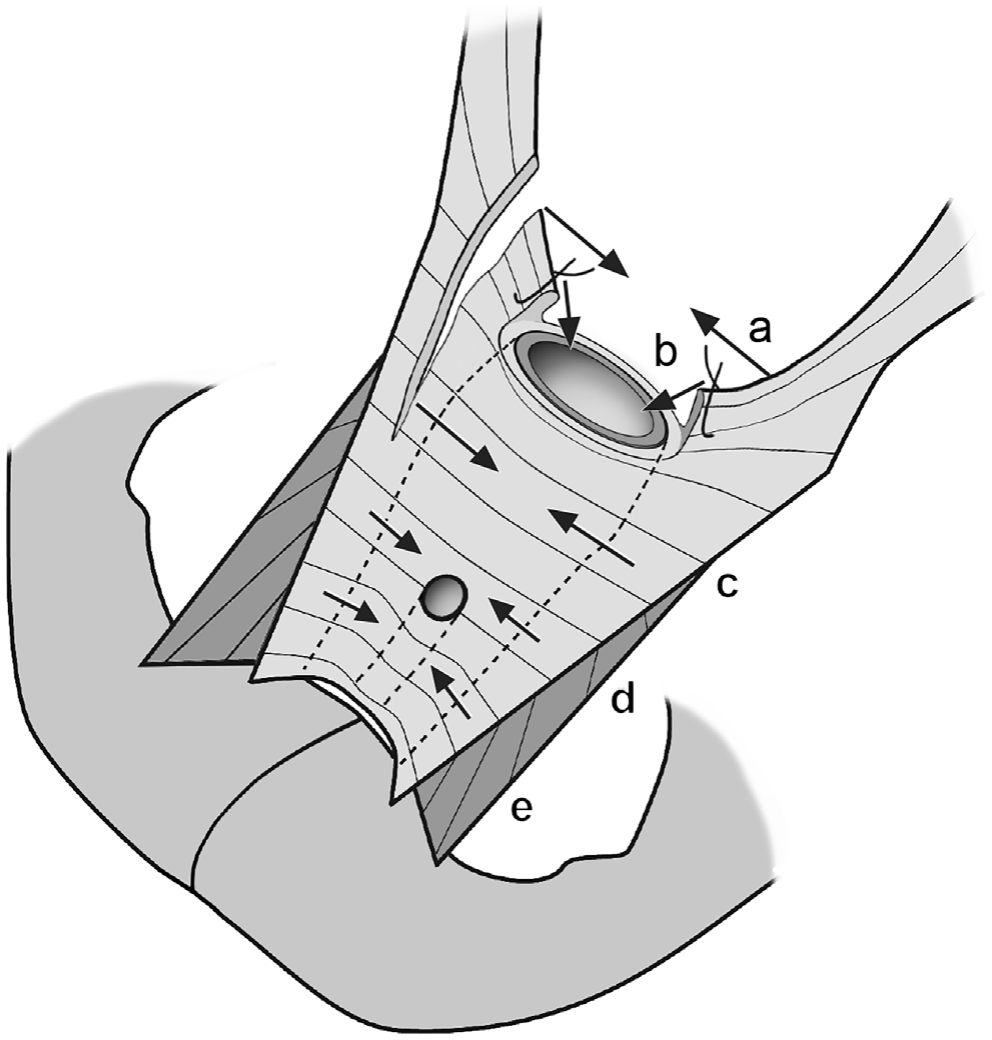
Principles of reconstruction of visceral pelvic fascia after vaginal hysterectomy. (a) McCall suture connects the remnants of the uterosacral ligaments above the vagina. (b) Stumps of the uterosacral and cardinal ligaments are mounted into the remnants of the pericervical ring. (c) Plication of the neighboring vesicovaginal and rectovaginal fascia reduces its potential laxity and begins repair of level II. (d) Kelly stitches. (e) Barnett stitches (suburethral buttress).

The suture can be placed at various heights above the vagina, using low or high McCall stitches. This choice depends on the extent of the defect. This technique also compensates for contralateral damage (Figure 4). Omitting this level significantly decreases the capacity of the surgery to relocate the apex and gives it adequate future stability. During vaginal hysterectomy, the stumps of the uterosacral and cardinal ligaments should be mounted to the tissue of the original pericervical ring. Using the outer one‐fourth of the apical suture is optimal, which should be arranged in a transverse direction (panel b in Figure 4). The repair of level II (see below) should then follow.

### 2.4 Level II

Vertical and horizontal mattress stitches correct a pulse component of cystocele or rectocele. A purse‐string stitch can also be helpful in some instances. Attachment to the structures of level I treats the apical component of cystocele and rectocele. On the anterior vaginal wall, relocation of the urethrovesical junction (UVJ) and support to the urethra can be achieved. Several techniques place additional sutures at the level of the proximal urethra (Kelly) or even the middle urethra and reinforce the tissue there (suburethral buttress stitches—Barnett) (panel d in Figure 4). These techniques were widely used before the era of suburethral slings. Their long‐term efficacy for stress urinary incontinence (SUI) is limited, but they can still be used to support the bladder and urethra before a suburethral sling is placed.

On the posterior vaginal wall, these stitches reduce the rectocele and are essential in the surgical treatment of obstructed defecation syndrome (ODS), which occurs when stool is retained in the pouch of the rectocele and cannot be expelled quickly during defecation. Repair of the visceral pelvic fascia at this level is effective in treating this bothersome disorder.^7^ Further, by reducing the redundant vaginal skin and tightening the underlying fascia, the width of the vagina is decreased. If it is well‐tailored, it helps maintain contact between the penis and vagina and improves the quality of sex for both partners. Midline plication does not improve a lateral defect directly.^8^ However, it still helps stabilize the tissue, with support from the contralateral side and upper and lower neighboring tissue. Specific techniques have been developed to address lateral defects at this level vaginally (vaginal paravaginal repair). Its universal feasibility and efficacy remain to be demonstrated.

### 2.5 Level III

Identifying parts of the perineal membrane with intact attachments to the pelvic bone is paramount. The reconstructed perineal body closes the urogenital hiatus with a solid, durable mass and restores the average distal end of the vaginal axis. This measure prevents future overloading of levels I and II. Torn edges of the perineal membrane after a rupture fall to the sides and detract some of the vaginal length. Its restoration adds up to several centimeters to the total vaginal length.

According to Delancey, level III also includes the superficial perineal space, comprising the bulbocavernosus muscle, superficial transverse perineal muscle, vaginal vestibule, and perineal skin, with its subcutaneous connective tissue.^5^ By repairing these structures, the vaginal introitus, vulva, and perineum regain their normal contour and appearance. The sealing effect of this apparatus is critical about continence, hygiene, and everyday female quality of life. Thus, its repair should not be neglected or viewed merely as an aesthetic measure.

For practical reasons, it would be helpful to categorize level III into 2 subsets: IIIA would include superficial structures (vaginal vestibule, perineal skin, subcutaneous tissue, bulbocavernosus muscle, and its attachment to the perineal body), and IIIB, which would comprise the perineal body itself (Figure 6). IIIA is often the focus of aesthetic procedures, primarily to correct scar tissue due to episiotomy or the width of the vaginal introitus or to reduce overstretched or discolored skin. Isolated repair of IIIA can be beneficial but is rarely functionally satisfactory in the presence of a IIIB defect. Conversely, paying attention only to IIIB without addressing IIIA is inadvisable. Both levels should be considered and adequately repaired.

### 2.6 Level IV

**FIGURE 5:**
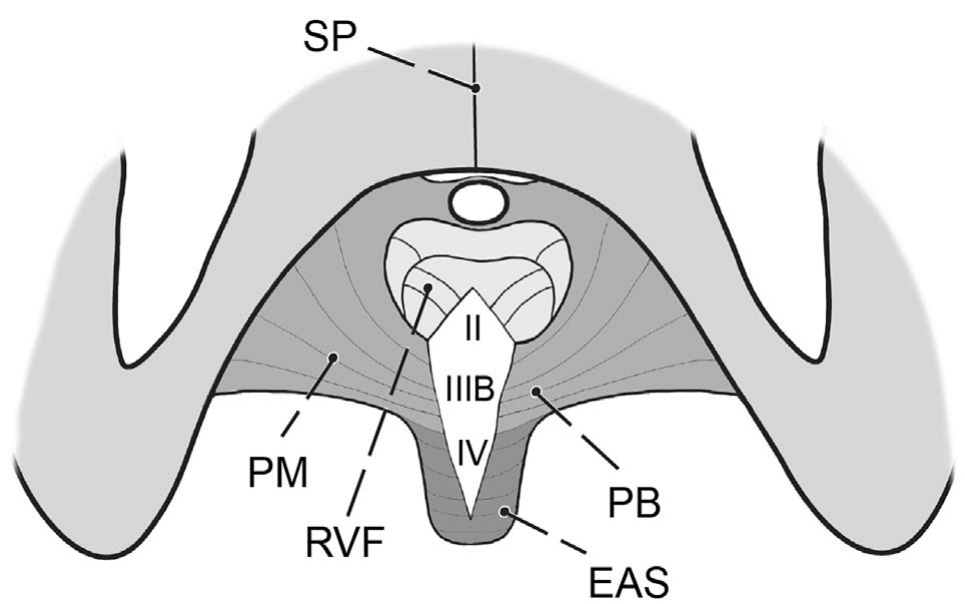
Schematic of perineal membrane, sphincter ani, and rectovaginal fascia. Common midline injury from vaginal childbirth, comprising the VPF (level II—rectovaginal fascia), perineal membrane (level III B—perineal body), and external anal sphincter muscle (level IV). EAS, external anal sphincter; PB, perineal body; PM, perineal membrane; RVF, rectovaginal fascia (= rectovaginal part of the visceral pelvic fascia (VPF); SP, symphysis pubis.

**FIGURE 6:**
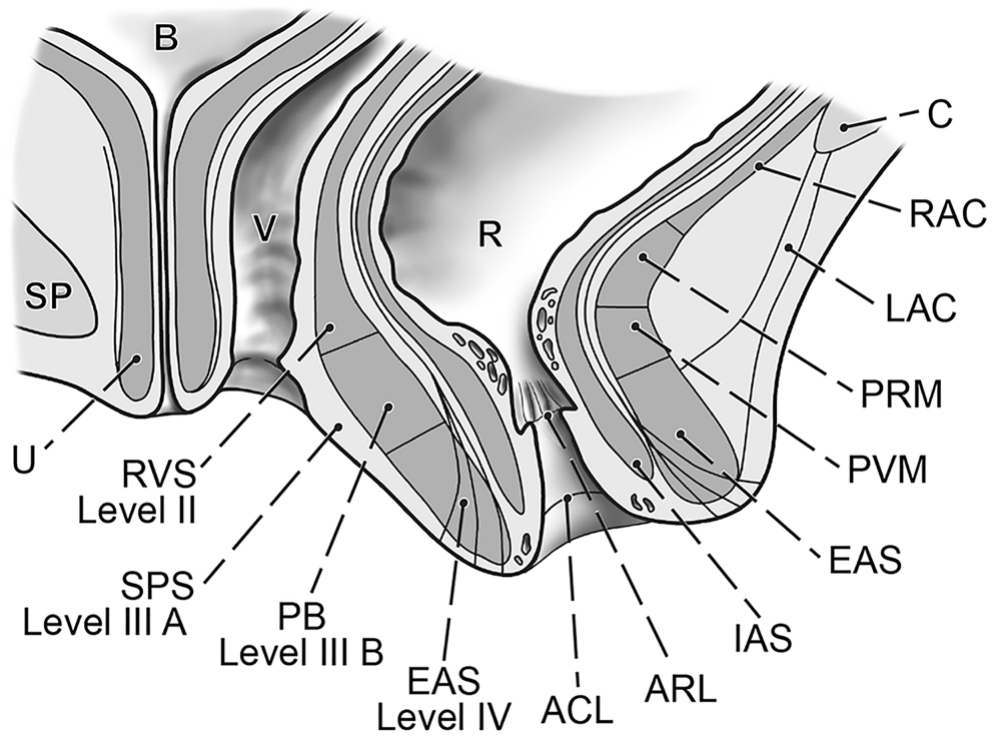
Schematic of sagittal view of the lower pelvic floor. Detailed view of the anal canal, sphincter ani muscle, and its relation to the levator ani muscle and rectovaginal septum. ACL, anocutaneous line (Hilton); ARL, anorectal line (dentate line); B, urinary bladder; C, coccyx; EAS, external anal sphincter; IAS, internal anal sphincter; LAC, anococcygeal ligament; PRM, puborectal muscle; PVM; pubovisceral muscle; RAC, anococcygeal raphe (also iliococcygeal raphe); R, rectum; RVS, rectovaginal septum; SP, symphysis; SPS, superficial perineal space; U, urethra; V, vagina.

The relation of the perineal body to the anal sphincter muscles is close (Figure 6). Moreover, these structures are commonly injured simultaneously during vaginal childbirth to various extents (Figure 5). The quality of direct adaptation and the healing process adds to the final pattern. To understand the presentation, tissue mapping with landmarks is useful. We check for continuity of the hymenal ring, vaginal vestibule, perineal skin, anal verge, anocutaneous line (border of the skin to the paler anoderm), and anorectal line (edge of the anoderm to the cylindrical epithelium of the rectum). Rectal examination and ultrasonography provide information on the external and internal sphincter muscles and their function.

**FIGURE 7:**
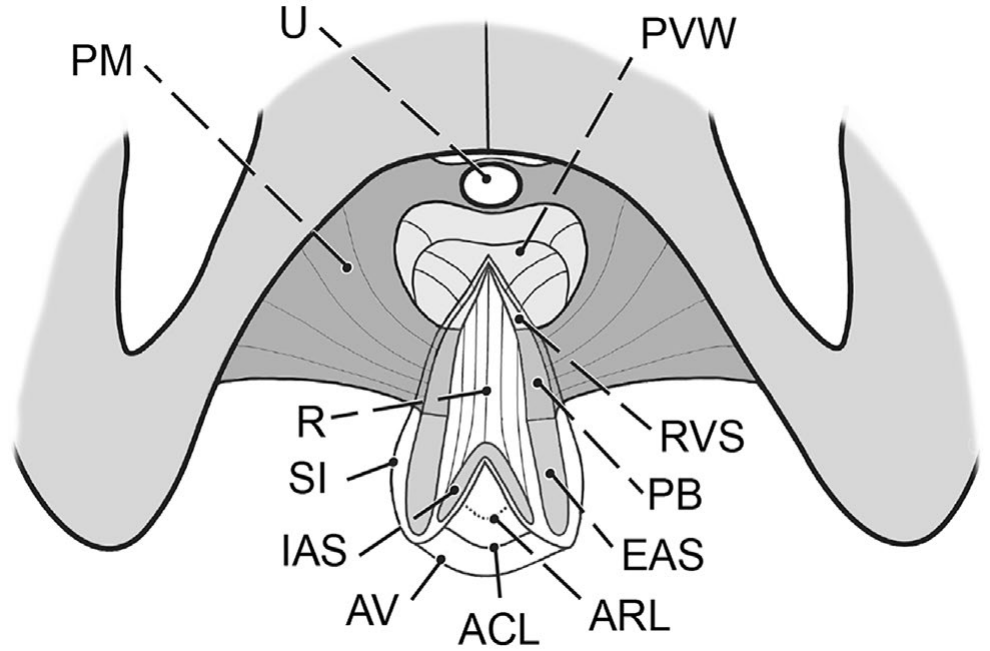
Complete perineal rupture after excision of the scar and mobilization of the involved structures. ACL, anocutaneous line (Hilton); ARL, anorectal line (dentate); AV, anal verge; EAS, external anal sphincter; IAS, internal anal sphincter; PB, perineal body; PM, perineal membrane; PVW, posterior vaginal wall; R, rectum and anal canal‐anterior wall; RVS, rectovaginal septum; SI, surgical incision (retracted, lambda shape); SP, symphysis pubis; U, urethra.

When a repair is needed, we primarily excise the old scars, identify the torn structures, and adapt them end to end. After an extensive anal tear, the scar often assumes a lambda shape. Its excision gives us adequate access to all involved structures (Figure 7).

## 3 DISCUSSION

Native tissue repair offers a robust set of techniques, which obtain high patient satisfaction when adequately chosen and performed.^2^, ^9^, ^10^ However, it still has limitations. We cannot repair a tear in the levator ani muscle which, if it is significant, severely disrupts the normal function of the entire pelvic floor. Specifically, defective muscle support to the connective tissue of the visceral pelvic fascia renders it prone to chronic overload and tissue elongation, thus increasing the likelihood of clinical recurrence. Collagen abnormality and chronic overload (obesity, difficult manual labor, poor compliance with physiotherapy) are additional major risk factors for recurrence.

In the case of significant fascial defects, tissue retrieval, which would serve for solid repair, might be difficult or even impossible. Newly sutured connective tissue might also remain weak and elongate sooner or later. Meticulously chosen reinforcement with synthetic material, such as polypropylene meshes, can be helpful in many such cases. More research is urgently needed in this field.

Compound or cascade solutions can help combine the strengths of various techniques. For example, laparoscopic or laparotomic mesh sacropexy for a significant level I defect, followed by vaginal repair of other levels, is an option. Sacropexy per se does not treat sexual dysfunction due to a wide vagina, obstructed defecation syndrome, or aesthetic issues and does not support the urethra. However, without solid support to the level I, the long‐term stability of the entire complex is unknown. Thus, a complementarity of techniques should be sought. Similarly, major lateral defects that are repaired with abdominal paravaginal defect repair can be combined with vaginal repair effectively. After successful repair, a mid‐urethral sling can be used in cases of urinary stress incontinence.

Adequate physiotherapy is critical for avoiding future excessive pressure load that might result in repeated secondary defects. It should always be included in the complex treatment of any pelvic floor disorder.

## 4 CONCLUSION

Native tissue repair is suitable for treating the highly variable pattern of defects of the female pelvic floor. Its limitations are attributed primarily to the surgeon's operative skills, the general condition of existing tissues, and future burdens. Although complete or total repair might not be achievable in all cases, the best possible restoration of anatomical structures can significantly improve many functional aspects of women's overall quality of life.

## AUTHOR CONTRIBUTIONS

Michal Otcenasek: Focus urogynecology first author, manuscript and diagrams preparation. Katarzyna Borycka: Focus proctology, manuscript preparation and literature review. Hynek Herman: focus obstetrics, senior and corresponding author, manuscript preparation.

## FUNDING INFORMATION

None.

## CONFLICT OF INTEREST STATEMENT

The authors have no conflicts of interest.

## Data Availability

Data sharing is not applicable to this article as no new data were created or analyzed in this study.
